# Varying doses of evening caffeine ingestion have different effects on rowing ergometer performance, sleep quality, and wakefulness scores

**DOI:** 10.3389/fnut.2025.1659220

**Published:** 2025-12-16

**Authors:** Izzet Karakulak, Ulaş Can Yildirim, Dilara Erkan, Raci Karayigit, Ender Eyuboglu, Azize Bingol Diedhiou, Mehmet Can Gundem, Selin Yildirim Tuncer, Halit Sar, Gokmen Ozen, Fırat Akca

**Affiliations:** 1 Department of Sport Management, Faculty of Sport Sciences, Mardin Artuklu University, Mardin, Türkiye; 2 Department of Coaching Education, Faculty of Sport Sciences, Sinop University, Sinop, Türkiye; 3 Department of Physical Education and Sports, Institute of Health Sciences, Faculty of Sport Sciences, Ankara University, Ankara, Türkiye; 4 Department of Coaching Education, Faculty of Sport Sciences, Ankara University, Ankara, Türkiye; 5 Department of Coaching Education, Faculty of Sport Sciences, Bartın University, Bartın, Türkiye; 6 Department of Coaching Education, School of Physical Education and Sports, Şırnak University, Şırnak, Türkiye; 7 Department of Sport Management, Faculty of Sport Sciences, Sinop University, Sinop, Türkiye; 8 Department of Coaching Education, Faculty of Sport Sciences, Lokman Hekim University, Ankara, Türkiye; 9 Department of Physical Education and Sports, Faculty of Sport Sciences, Sinop University, Sinop, Türkiye; 10 Department of Physical Education and Sports Education, Faculty of Sport Sciences, Çanakkale Onsekiz Mart University, Çanakkale, Türkiye

**Keywords:** caffeine, dose-response, rowing performance, sleep quality, wakefulness

## Abstract

**Introduction:**

This study investigated the dose-dependent effects of evening caffeine ingestion on rowing performance, sleep quality, and daytime sleepiness in trained male rowers.

**Methods:**

Using a double-blind, randomized, crossover design, 13 university-level rowers (mean age = 22.07 ± 2.21 years; mean body mass = 77.66 ± 6.45 kg) completed four 2,000 m time–trial sessions between 19:00 and 20:00 h under placebo (PLA), low-dose capsule caffeine (3 mg/kg, LDC), moderate-dose capsule caffeine (6 mg/kg, MDC), and high-dose capsule caffeine (9 mg/kg, HDC) conditions. Performance metrics, heart rate, and subjective sleep assessments were collected. Rowing performance was assessed by a standard 2,000 m rowing ergometer (Concept II, United States) time trial. Sleep quality was measured with a numerical rating scale in the morning after each trial, and daytime sleepiness was measured with the Karolinska Sleepiness Scale.

**Results:**

Results indicated significantly improved rowing times and power output with HDC and MDC compared to PLA (*p* < 0.05), with HDC yielding the most notable enhancements (*d* = 0.40–0.41). However, these ergogenic benefits were accompanied by significantly impaired sleep quality and elevated daytime sleepiness in both HDC and MDC groups (*p* < 0.01; *d* = 1.3–1.5). Notably, adverse effects such as headache, insomnia, and gastrointestinal discomfort were predominantly reported in the HDC condition (*p* < 0.05). Although LDC offered mild performance improvements with minimal sleep disruption, only the high dose condition exhibited large physiological and perceptual trade-offs.

**Discussion:**

These findings indicate a clear dose–response relationship, wherein higher evening caffeine intake improves performance but has detrimental effects on sleep and recovery markers. Coaches and athletes should carefully balance caffeine dosing against potential recovery costs, especially in evening training or competition contexts.

## 1 Introduction

The use of caffeine, a component of the methylxanthine group, as an ergogenic aid is widely preferred by athletes to increase physical and cognitive performance (1). More than 75% of athletes in various sport disciplines take caffeine supplements to gain an advantage before or during competition (2, 3). Caffeine use has been reported to increase, especially in individual and aerobic-based sports. Additionally, among the analyzed athletes, rowers appear to be among the highest caffeine users (4).

The desired form of caffeine can be chosen, including coffee, capsule, gum, bar, gel and aerosol (5). Acute caffeine intake has been confirmed to have a positive effect on athletic performance related to parameters such as cardiovascular endurance (6), anaerobic endurance (7), movement speed (8), power (9) and muscular endurance during resistance exercises (10). The most widely known mechanism of caffeine’s ergogenic effects is through antagonism of adenosine receptors, which in turn reduces the concentration of several central nervous system neurotransmitters, including serotonin, dopamine, acetylcholine, norepinephrine, and glutamate (11). Caffeine compounds inhibit the known effect of adenosine by binding to adenosine receptors due to their similar structure to adenosine. It is known to improve aerobic-muscle endurance performance by increasing the release of neurotransmitters that promote wakefulness (12–15). This effect may help maintain performance by supporting the maintenance of neural stimulation in high-exertion sports like rowing. Indeed, sustained neural activation and a reduction in perceived exertion have been reported to enhance performance during prolonged efforts with caffeine supplementation (16). In endurance athletes, caffeine intake is known to maintain muscle force production and reduce the perception of fatigue through central nervous system stimulation (17).

Rowing is a sport that is likely to be affected by natural circadian (24-h) or diurnal (time of day) changes because significant muscle strength is required especially in the drive phase of the rowing stroke (18). In a study supporting this view, in which performances on the 2,000 m rowing ergometer were evaluated in the morning and evening, it was reported that, regardless of chronotype, athletes performed an average of 2.4 s faster in the morning training compared to the evening training (19). This finding supports researchers’ view that caffeine’s ergogenic effects can be used to offset performance declines that may occur in the evening. Therefore, caffeine may offer a potential advantage, particularly against performance declines that may occur later in the day. However, despite its potential to improve performance, caffeine can cause side effects such as headache, nausea, insomnia or anxiety (20). In a meta-analysis examining the side effects that occur in athletes after caffeine supplementation, it was reported that there was a 34% higher probability of side effects after consuming low and moderate doses of caffeine (21). The same meta-analysis study emphasized that heart palpitations and sleep problems were the most frequently reported side effects. Evening use of caffeine, in particular, may increase exposure to adverse effects on sleep due to caffeine’s long half-life (22). Caffeine (200 mg, 3+3 mg/kg, 6 mg/kg), especially when taken in the afternoon, both before and after exercise, is known to disrupt sleep-related parameters (23–25). Considering the negative effects of sleep deprivation on exercise performance (26, 27), it is important to identify the conditions that pose a risk to athletes’ sleep quality. The effects of afternoon caffeine intake on sleep efficiency the following night have been the focus of some researchers. Moderate doses (6 mg/kg) of caffeine taken before cycling exercises at approximately 5:00 p.m. delayed falling asleep and shortened total sleep time (24). Another similar study reported that 6 mg/kg of caffeine taken before an 800-m running test at 8:00 p.m. reduced sleep efficiency (25). In the same study negative feedback was received from athletes for subjective sleep parameters such as “sleep quality,” “calm sleep,” “ease of falling asleep,” and “feeling refreshed after waking”. Other studies involving rugby players have also found negative or neutral findings on caffeine-related sleep quality (28, 29) In the study conducted by Caia et al. (28) post-competition salivary caffeine concentrations of athletes who continued their usual caffeine consumption before or during the competition were analyzed. A moderate negative relationship was found between the increase in caffeine levels and sleep onset delay and sleep efficiency. This effect of caffeine on sleep efficiency is attributed to the time it takes to be metabolized. After caffeine is taken, its concentration in the blood plasma typically reaches its highest level within 60 min and it takes approximately 4–6 h for half of the initial dose to be metabolized (30). Therefore, caffeine consumed before going to bed reduces sleep duration and sleep efficiency (31). In addition, it can be said that caffeine reduces sleep efficiency due to the tendency of the individual to temporarily increase the number of awakenings during the sleep period (32). Given that rowing training is often performed in the evening, such sleep disruptions may negatively impact recovery processes and reduce performance capacity the next day. Therefore, total sleep time will also decrease.

There are conflicting studies on the optimum caffeine dose in caffeine consumption (17, 33). Although the optimal dose required for the ergogenic effect of caffeine varies depending on gender (34), muscle group size (35), and habitual caffeine consumption (36), research generally shows that it improves exercise performance when consumed at doses of 3–6 mg/kg (37, 38). Moderate to high doses (6–9 mg/kg) of caffeine significantly improve short-duration, high-intensity rowing performance by reducing the time required to complete the distance on a 2,000 m rowing ergometer (39). Similarly, 6 mg/kg of caffeine is observed to significantly improve mean power output in 2,000 m rowing performance (40). Contrary to these findings, in the study conducted by Skinner et al. (41), it was concluded that 2, 4, and 6 mg/kg of caffeine did not provide any improvement in performance. The conflicting results regarding caffeine and rowing performance suggest that further randomized studies are needed. This is supported by a study conducted by Filip-Stachnik et al. (42) on judokas. After consuming 3 mg/kg of caffeine in the evening (19:00), they concluded that low-dose caffeine did not cause a significant deterioration in objective sleep parameters after the following night’s sleep analysis of athletes who trained. However, the lack of studies in the literature that evaluate the effects of caffeine use on performance, as well as the effects on sleep quality the night following use and the level of alertness the next day, is striking. In addition, randomized studies in the literature where different doses are observed in the same sample group are limited. Moreover, verification of the effect of different caffeine doses on sleep quality will provide valuable information especially for athletes who use caffeine before evening training or competitions (43). Finally, considering dose-dependent side effects, low-dose caffeine supplementation ( ≤ 3 mg/kg) has been reported to both enhance athletic performance and carry a relatively low risk of side effects (17). Therefore, the negative effects of low doses on sleep quality are likely minimal.

In this context, the aim of the present study was to examine the effects of different doses of caffeine taken in the evening on (1) ergometric rowing performance, (2) sleep quality, and (3) alertness levels. This research raised the following hypotheses: Low, moderate, and high doses of caffeine ingested in the evening will dose-dependently affect rowing ergometer performance (completion time and average power output), subjective sleepiness levels, and subsequent sleep quality. Higher doses are also expected to enhance performance but impair sleep parameters.

## 2 Materials and methods

### 2.1 Study design

The study followed a double-blind, within-subjects, fully counterbalanced 4 × 4 Latin square design to control for order and sequence effects. Each participant (*n* = 13) completed all four conditions; placebo (PLA), low dose caffeine (LDC; 3 mg/kg), moderate dose caffeine (MDC; 6 mg/kg), and high dose caffeine (HDC; 9 mg/kg) in a unique random order. Randomization was conducted by an independent research assistant, uninvolved in participant recruitment or data collection, using (44), a publicly accessible online random sequence generator. For each participant, a 4 × 4 Latin square was generated with the constraint that each condition appeared exactly once in each trial position (1st, 2nd, 3rd, 4th) across the full sample. The resulting 13 unique sequences ensured complete counterbalancing. The experimental trials were scheduled with a minimum interval of 72 h and a maximum of 7 days between sessions to ensure sufficient treatment washout, adequate participant recovery and minimize carry-over effects (45, 46). Carry-over effects were assessed by including testing order as a factor in the linear mixed model; no significant main effects or interactions were found (all *p* > 0.40), confirming the adequacy of the washout period and counterbalancing. A research assistant blinded to the randomization list enrolled participants and obtained informed consent. Allocation concealment was ensured via sequentially numbered, opaque, sealed envelopes (SNOSE). Envelopes were opened only after baseline assessments were completed. Both participants and the outcome assessor were blinded to group assignment. The intervention administrator prepared caffeine/placebo doses in identical capsules labeled only with participant codes. Blinding was maintained until statistical analysis was finalized.

**FIGURE 1:**
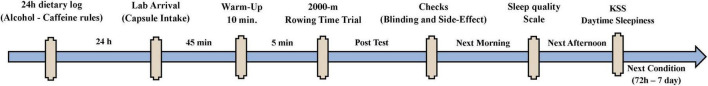
Test protocol. h, hour; lab, laboratory; min, minutes; KSS, Karolinska Sleepiness Scale; m, meters.

Every time trial was performed using a Concept II Model D Rowing ergometer (Concept II, Morrisville, VT, United States), with both the completion time and average power output (measured in watts) being documented. Additionally, heart rate measurements were taken during the tests utilizing a Polar Team Pro system equipped with an H10 sensor (Polar Electro OY, Kempele, Finland). The experimental trials (Figure 1) were scheduled with a minimum interval of 72 h and a maximum of 7 days between sessions to ensure sufficient treatment washout and adequate participant recovery (45).

Each participant’s warm-up routine was documented during the initial trial and then consistently reproduced in the subsequent trials. Testing sessions were uniformly scheduled in the evening, between 19:00 and 20:00, and at identical times for each participant to mitigate the influence of circadian rhythms. For each experimental trial, participants were asked to report any side effects they might have encountered.

### 2.2 Participants

The investigation involved a group of 13 male university-level rowers, with an average age of 22.07 years [standard deviation (SD) = 2.21], body mass of 77.66 kg (SD = 6.45), height of 182.14 cm (SD = 7.11), body fat percentage of 11.23% (SD = 4.1), and a typical daily caffeine consumption of 303.62 mg (SD = 148.34). These individuals had accrued an average of 3.1 years (SD = 1) of rowing training experience.

The required sample size was determined using G*Power software (version 3.1.9.4; Dusseldorf, Germany), based on an analysis of variance (ANOVA) design incorporating repeated measures and within-subjects factors. The calculation utilized an effect size (f) of 0.25, a significance level (alpha) of 0.05, and a statistical power of 0.95 and *r* = 0.85 with a single cohort of participants. This target effect size was chosen as a medium effect per Cohen (47) and is consistent with effect sizes reported in prior studies examining the ergogenic effects of caffeine on 2,000-m rowing performance [e.g., f ≈ 0.20–0.28; (41, 48)]. It was detected that at least 12 participants were needed for adequate power, we included work with 13 participants to stay in the safe side if any injury etc. occurs.

During their initial laboratory visit, participants underwent anthropometric assessments. Height and body mass were recorded using a Seca stadiometer (Seca Deutschland, Hamburg, Germany), while body fat percentage was evaluated with an InBody 770 body composition analyzer (InBody Co., Gangnam-Gu, Seoul, Korea). Following these measurements, participants were provided with detailed study protocol information sheets and signed informed consent forms.

Eligibility for participation was restricted by specific exclusion criteria. Rowers were excluded from the study if they presented with a medical condition that impaired their capacity to follow the study protocol, current use of prescription medications, a confirmed allergy to mannitol or any other sweeteners, a diagnosed sleep disorder; or a physician’s recommendation to limit or avoid caffeine intake. Participants were eligible for the study if they met the following criteria: competitive rowers with a minimum of 2 years of structured training experience and currently training ≥ 10 h per week; aged 18–35 years-male; generally healthy, with no chronic medical conditions and injuries for the last year; and habitual caffeine intake classified as moderate (100–400 mg/day, self-reported via a food diary prior to enrolment).

### 2.3 Supplementation protocol

Capsules, identical in appearance, were used to deliver the substances for all conditions. The dosages of caffeine were individually calculated based on each participant’s weight. Placebo capsules were filled with sugar alcohol (mannitol), not expected to have any further effects on performance (49). Caffeine capsules contained caffeine powder. In both caffeine and placebo experiments, participants consumed 3–5 gelatin capsules, with the quantity of capsules standardized across all subjects. Researchers and participants could not distinguish between placebo and caffeine capsules due to equal color and size. At the end of each trial, participants were asked to indicate their assumption regarding the type of capsules swallowed. The capsules were provided 60 min before each trial in order to have sufficient time to ensure increasing blood caffeine levels (35). The supplementation groups were as follows; 3 mg/kg caffeine (low dose caffeine, LDC), 6 mg/kg caffeine (moderate dose caffeine, MDC), 9 mg/kg caffeine (high dose caffeine, HDC) or placebo (PLA). Prior to departing the laboratory, participants were queried regarding their perception of the treatment administered, specifically whether they believed they had received no caffeine, a low dose, a moderate dose, or a high dose. Furthermore, physical exhaustion and adverse effects (such as gastrointestinal issues, tachycardia, muscle pain, or headaches) experienced 24 h post-supplement administration were documented via an online questionnaire. The questionnaire comprised eight binary (yes/no) items, adapted from prior studies examining the adverse effects associated with caffeine consumption ((35). Owing to the physically demanding nature of the rowing time trial, the collection of side effect responses was scheduled exclusively for 24 h post-test.

### 2.4 Diet and caffeine consumption control

The research participants were instructed to abstain from alcohol consumption and vigorous physical training for a 24-h period preceding each experimental session. Throughout the duration of the study, they were advised to refrain from using any dietary supplements. All participants were required to maintain a detailed 24-h dietary record on the day prior to each testing session, as well as a weekly log of their caffeine intake. To ensure consistency in energy consumption and hydration status, participants were directed to replicate their dietary intake, as documented in the food log, before every trial. Daily caffeine consumption was quantified using a modified version of the questionnaire developed by Bühler et al. (50). Additionally, the caffeine content from various food and beverage sources was incorporated to determine the total daily caffeine intake. Based on this evaluation, all participants were classified as habitual moderate caffeine consumers, in accordance with the criteria established by Filip et al. (51). To simulate conditions reflective of real-world athletic environments, as recommended by Tallis et al. (52), participants were encouraged to maintain their usual daily caffeine intake throughout the study. This approach was implemented to mitigate the potential impact of caffeine withdrawal, as noted by Pickering and Kiely (46).

Compliance with these dietary and caffeine control instructions was verified prior to each session through direct review of the submitted 24-h dietary records and caffeine logs. Any discrepancies (e.g., alcohol intake, supplement use, or vigorous activity) were addressed by rescheduling the trial to ensure full adherence. To support dietary replication, participants were provided with a standardized copy of their initial food record for subsequent sessions, and adherence was confirmed by cross-checking macronutrient and caloric patterns across trials. No cases of non-compliance were detected.”

### 2.5 Subjective sleep quality and daytime sleepiness measurements

Participants were directed to maintain their normal sleep patterns both prior to and following the experimental sessions. Each morning subsequent to a trial, they assessed their sleep quality utilizing a validated numeric rating scale, with scores ranging from 0 (indicating “worst possible sleep”) to 10 (denoting “best possible sleep”) (53). Furthermore, levels of daytime sleepiness were evaluated in the afternoon through the application of the Karolinska Sleepiness Scale (54), which employs a numeric range from 1 (representing “extremely alert”) to 9 (signifying “very sleepy”). Both assessments were self-administered at home via a secure online platform (Research Electronic Data Capture, REDCap). Participants received automated email reminders each morning and afternoon with direct links to the questionnaires. Completion was mandatory to proceed to the next trial; adherence was verified by timestamped electronic submissions and confirmed 100% compliance across all participants.

### 2.6 2 ,000-m rowing time trial

The 2,000 m time trial was performed on the same rowing ergometer for each participant. The test-retest reliability of 2,000 m time trial on the Concept II rowing ergometer has previously been examined previously with well-trained rowers and was reported with a coefficient of variation (CV) of 0.6% (55). Each participant’s warm-up routine was documented during the initial trial and then consistently reproduced in the subsequent trials. The time to complete the time trial was recorded. The stroke rate during the test was freely selectable by each subject and the drag factor settings of the ergometers were adjusted to 140 as recommended by Amateur Rowing Association for heavyweight men rowers (56). During the 2k test, after a self-selected warmup, the athletes were required to row 2,000 m in the least time possible. This test is a standard criterion used for national team selection purposes in many countries (57, 58) and was performed routinely by all rowers in this study.

### 2.7 Statistical analysis

The normality of the dataset was initially assessed using the Shapiro-Wilk test. Following this, the sphericity assumption was evaluated with Mauchly’s test, and the Greenhouse-Geisser correction was implemented whenever violations of sphericity were evident. To examine differences in test completion time, heart rate, power output, and subjective sleep and daytime sleepiness parameters across the full duration of the testing period, a repeated measures analysis of variance (ANOVA) was employed. Relative change values were calculated to quantify the magnitude of differences between each caffeine condition and placebo. The relative change for each variable was computed using the following formula:


$$RelativeChange\left(\%\right)=\left[\frac{V\,a\,l\,u\,e_{C\,o\,n\,d\,i\,t\,i\,o\,n}-V\,a\,l\,u\,e_{P\,l\,a\,c\,e\,b\,o}}{V\,a\,l\,u\,e_{P\,l\,a\,c\,e\,b\,o}}\right]\times 100$$

The partial eta square (η*_*p*_*^2^) was utilized to assess the effect size, which was classified as small (0.10–0.24), moderate (0.25–0.39), or large (≥ 0.40). Where significant effects emerged, Bonferroni *post hoc* paired comparisons were conducted to identify specific differences between conditions. Cohen’s d effect sizes for repeated measures, reported alongside their 95% confidence intervals (95% CI). These effect sizes were interpreted according to the following benchmarks: values less than 0.20 were deemed trivial, those ranging from 0.20 to 0.49 were classified as small, 0.50–0.79 as moderate, and values of 0.80 or higher as large (59). Additionally, dichotomous side-effect responses (yes/no) were analyzed using Cochran’s Q test for overall differences across the four conditions. Pairwise comparisons were performed using McNemar exact tests. Odds ratios (OR) and 95% confidence intervals (CI) were calculated using the Haldane-Anscombe correction (adding 0.5 to zero cells) to handle zero counts. Significance was defined at *p* < 0.05. Statistical computations were executed using SPSS software (version 30; IBM Corp., Armonk, New York, United States), while data visualizations were created with GraphPad PRISM Software (Version 10.4, GraphPad Inc., San Diego, CA, United States).

## 3 Results

Mean values and relative (%) changes (compared to PLA) of all measured variables during rowing time trials have been presented in Table 1.

**TABLE 1:** Rowing time trial and sleep performance variables (mean ± SD) across supplementation conditions.

Parameters	*N* = 13	Mean	SD	Relative change (%) HDC compared to PLA mean (SD)	Relative change (%) MDC compared to PLA mean (SD)	Relative change (%) LDC compared to PLA mean (SD)
Time trial completion time (seconds)	PLA	407.585	10.615	1.03 (0.97)	0.75 (0.86)	0.48 (0.90)
	HDC	403.285	10.657			
	MDC	404.338	10.602			
	LDC	405.715	11.067			
Mean power output (watts)	PLA	331.962	25.874	3.21 (2.98)	2.33 (2.65)	1.49 (2.76)
	HDC	342.777	26.673			
	MDC	340.246	26.248			
	LDC	336.877	27.044			
Average heart rate (beats per minute)	PLA	183.077	3.451	0.71 (0.96)	0.46 (0.92)	0.35 (1.07)
	HDC	185.538	3.431			
	MDC	184.462	3.455			
	LDC	184.077	3.278			
Subjective sleep quality (SSQ)	PLA	7.000	0.707	13.83 (10.32)	11.45 (11.47)	8.06 (10.77)
	HDC	6.000	0.707			
	MDC	6.154	0.689			
	LDC	6.385	0.506			
Karolinska Sleepiness Scale (KSS)	PLA	3.231	0.725	33.59 (36.53)	27.95 (36.76)	12.31 (36.72)
	HDC	4.308	0.947			
	MDC	4.231	0.725			
	LDC	3.538	0.660			

PLA, Placebo; HDC, High Dose Caffeine; MDC, Moderate Dose Caffeine; LDC, Low Dose Caffeine.

As shown in Table 1, the mean ± SD values for all measured variables across the four conditions are presented together with the corresponding relative percentage changes compared with placebo. Briefly, time-trial completion time improved by 1.03, 0.75, and 0.48% in the HDC, MDC, and LDC conditions, respectively. Mean power output increased by 3.21, 2.33, and 1.49% across the same conditions. Average heart rate exhibited minimal relative elevations (0.71, 0.46, and 0.35% for HDC, MDC, and LDC, respectively). Subjective sleep quality decreased by 13.83, 11.45, and 8.06%, whereas Karolinska Sleepiness Scale scores increased by 33.59, 27.95, and 12.31% in HDC, MDC, and LDC compared with placebo.

**FIGURE 2:**
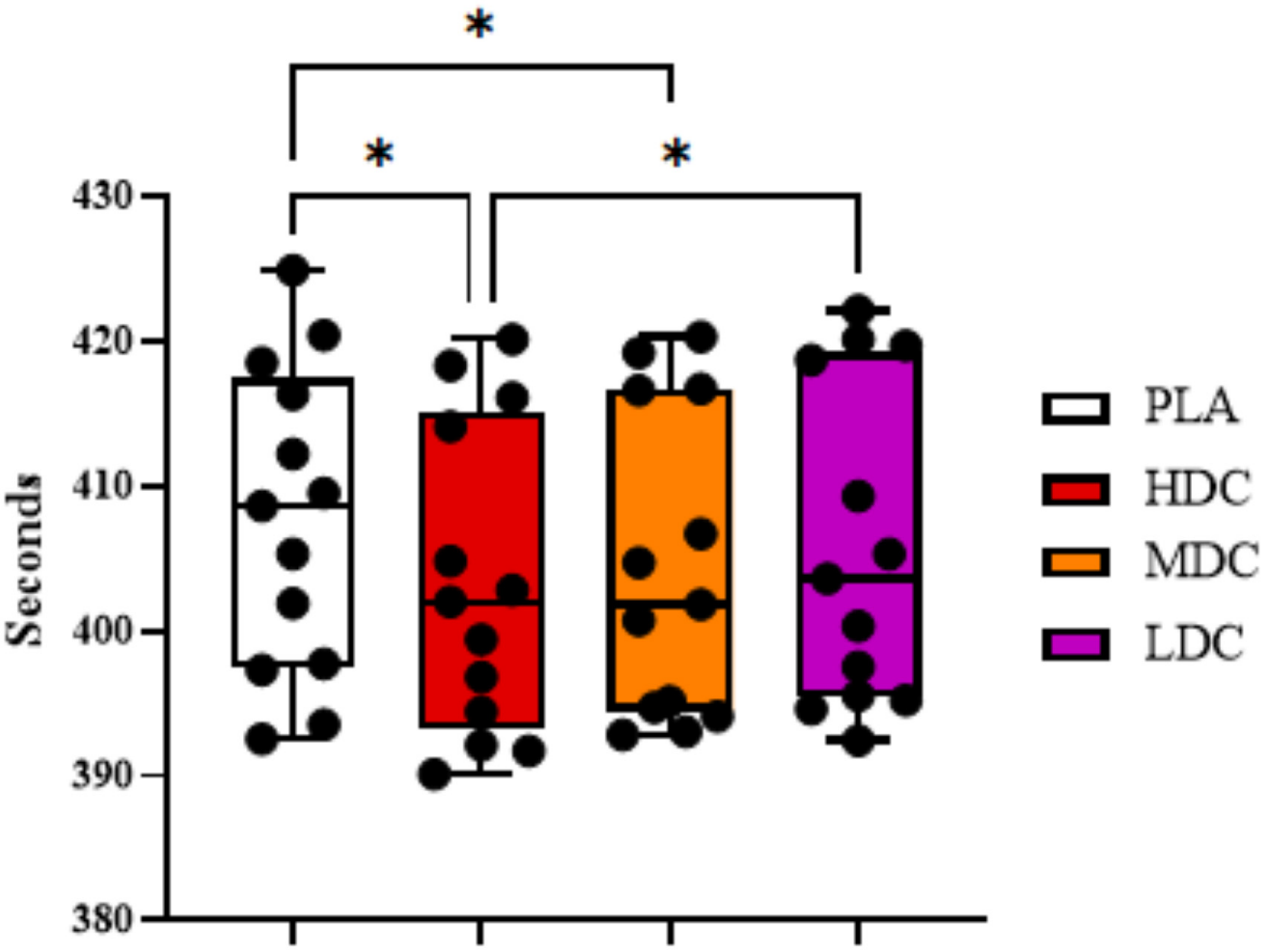
2,000 m rowing ergometer time trial completion time. PLA, Placebo; HDC, High Dose Caffeine; MDC, Moderate Dose Caffeine; LDC, Low Dose Caffeine. *p < 0.05.

Effects of different supplementations on rowing ergometer time trial time have been demonstrated in Figure 2.

There was a significant difference in rowing time trial performance between conditions (*p* = 0.001, η*_*p*_*^2^ = 0.45). Bonferroni *post hoc* analysis showed time trial completed faster in HDC condition (403.285 ± 10.657 s) than PLA (407.585 ± 10.615 s, *p* = 0.013; 95% CI = 0.807–7.793; *d* = 0.40). Time trial performance was significantly better in MDC (404.338 ± 10.602 s) condition compared to PLA (407.585 ± 10.615 s, *p* = 0.039; 95% CI = 0.136–6.356; *d* = 0.30). In addition, the results were significantly better for HDC condition (403.285 ± 10.657 s) compared to LDC condition (405.715 ± 11.067 s, *p* = 0.024; 95% CI = 0.271–4.590; *d* = 0.23).

**FIGURE 3:**
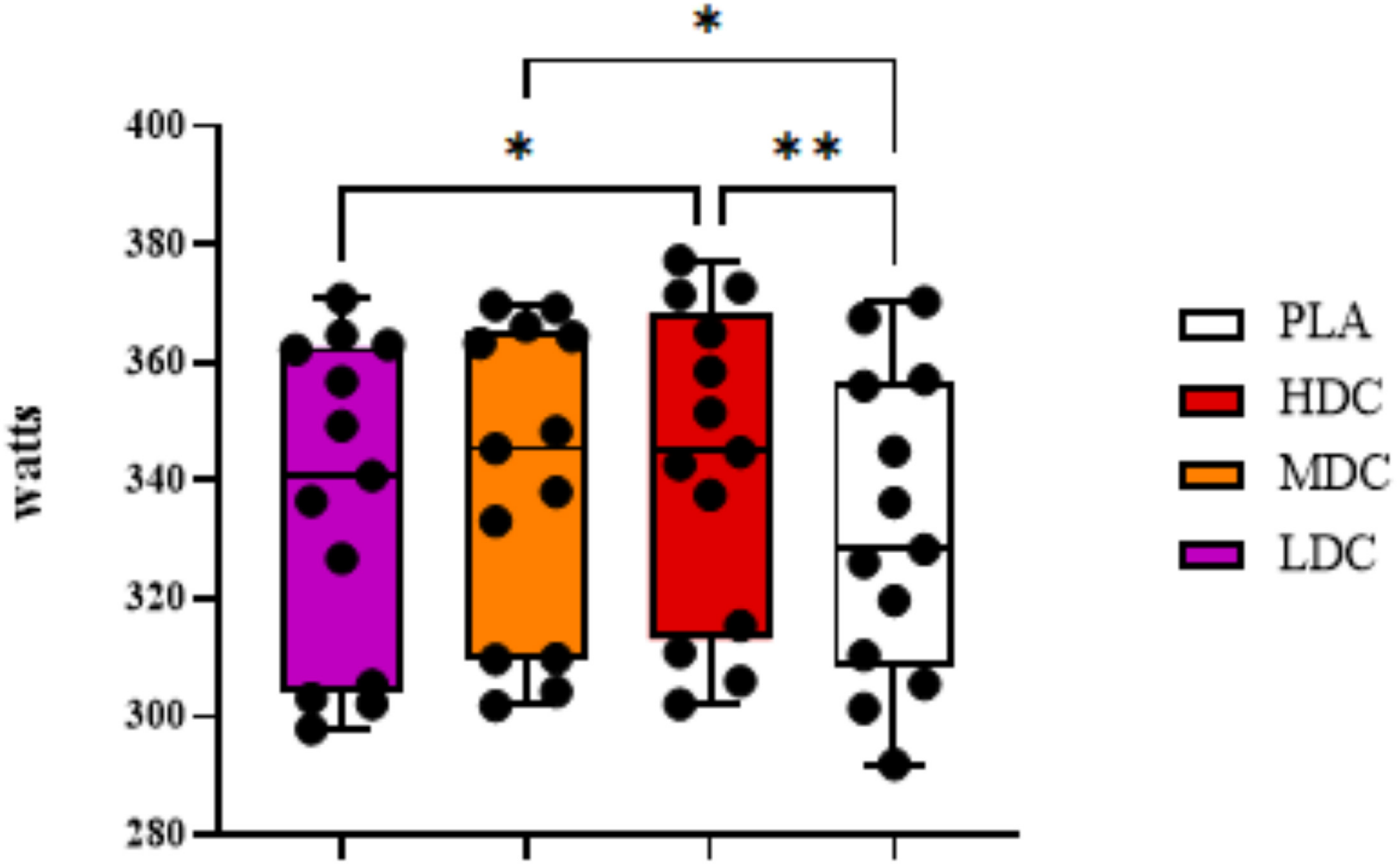
Mean power output during 2,000 m rowing ergometer time trial. PLA, Placebo; HDC, High Dose Caffeine; MDC, Moderate Dose Caffeine; LDC, Low Dose Caffeine. *p < 0.05, **p < 0.01.

Mean power output during Time trial has been provided in Figure 3.

There was a significant difference in time trial mean outputs between conditions (p = 0.001, η*_*p*_*^2^ = 0.47). Bonferroni *post hoc* analysis showed higher power outputs for HDC (342.777 ± 26.673 watts) compared to PLA (331.962 ± 25.874 watts, *p* = 0.009; 95% CI = 2.513–19.118; *d* = 0.41). The results were significantly better for HDC condition (342.777 ± 26.673 watts) compared to LDC condition (336.877 ± 27.044 watts, *p* = 0.028; 95% CI = 0.537–11.263; *d* = 0.23). In addition, higher power outputs for MDC (340.246 ± 26.248 watts) were detected compared to PLA (331.962 ± 25.874 watts, *p* = 0.027; 95% CI = 0.788–15.782; *d* = 0.31).

**FIGURE 4:**
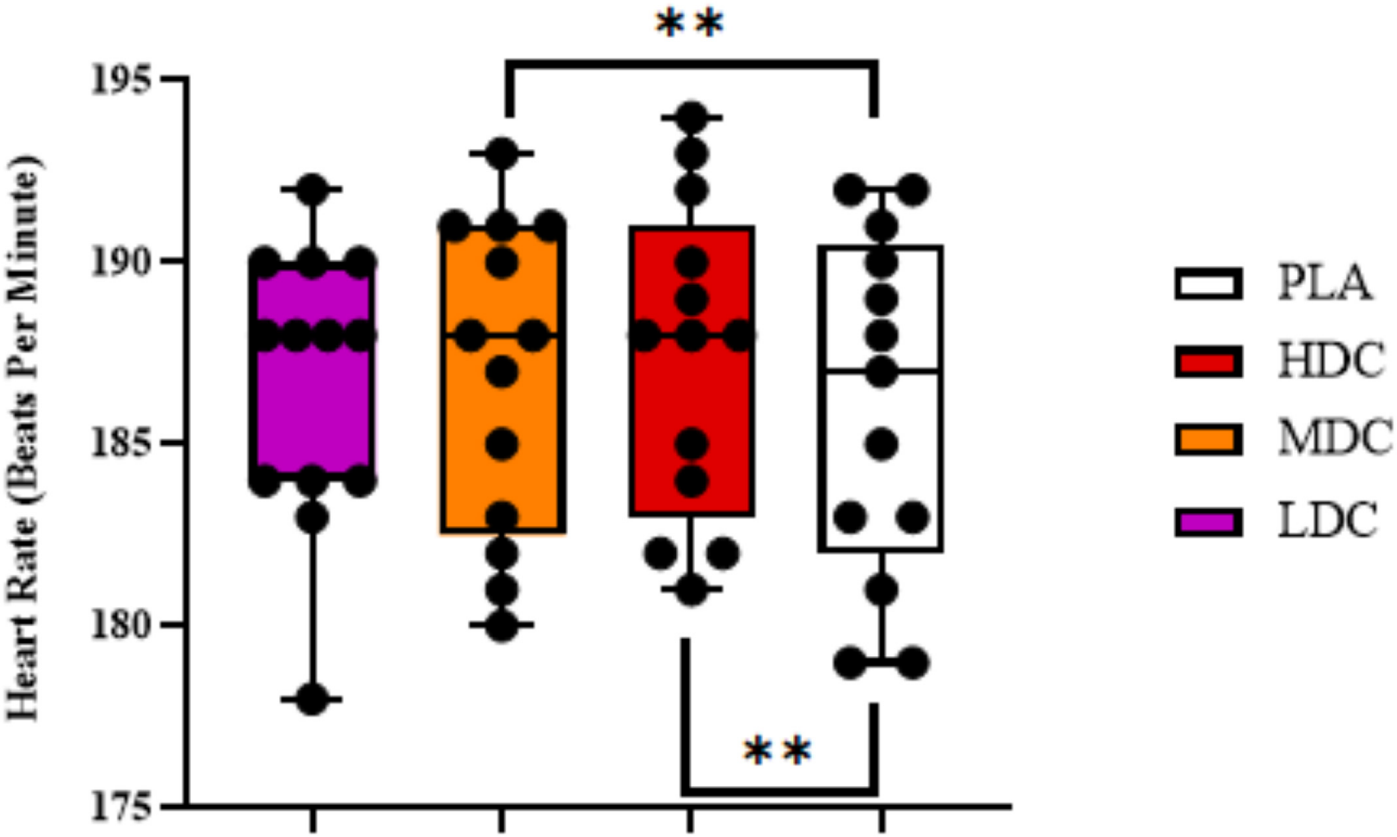
Average heart rate during 2,000 m rowing ergometer time trial. PLA, Placebo; HDC, High Dose Caffeine; MDC, Moderate Dose Caffeine; LDC, Low Dose Caffeine. **p < 0.01.

Average heart rate values obtained during time trial have been presented in Figure 4.

There was a significant difference in heart rate values between conditions (*p* = 0.001, η*_*p*_*^2^ = 0.56). Bonferroni *post hoc* analysis showed a higher heart rate for HDC [185.538 ± 3.431 beats per minute (bpm)] compared to PLA (183.077 ± 3.451 bpm, *p* = 0.002; 95% CI = 1.695–3.228; *d* = 0.72). In addition, the results were significantly different between MDC condition (184.462 ± 3.455 bpm) and PLA condition (183.077 ± 3.451 bpm, *p* = 0.005; 95% CI = 0.405–2.365; *d* = 0.40).

**FIGURE 5:**
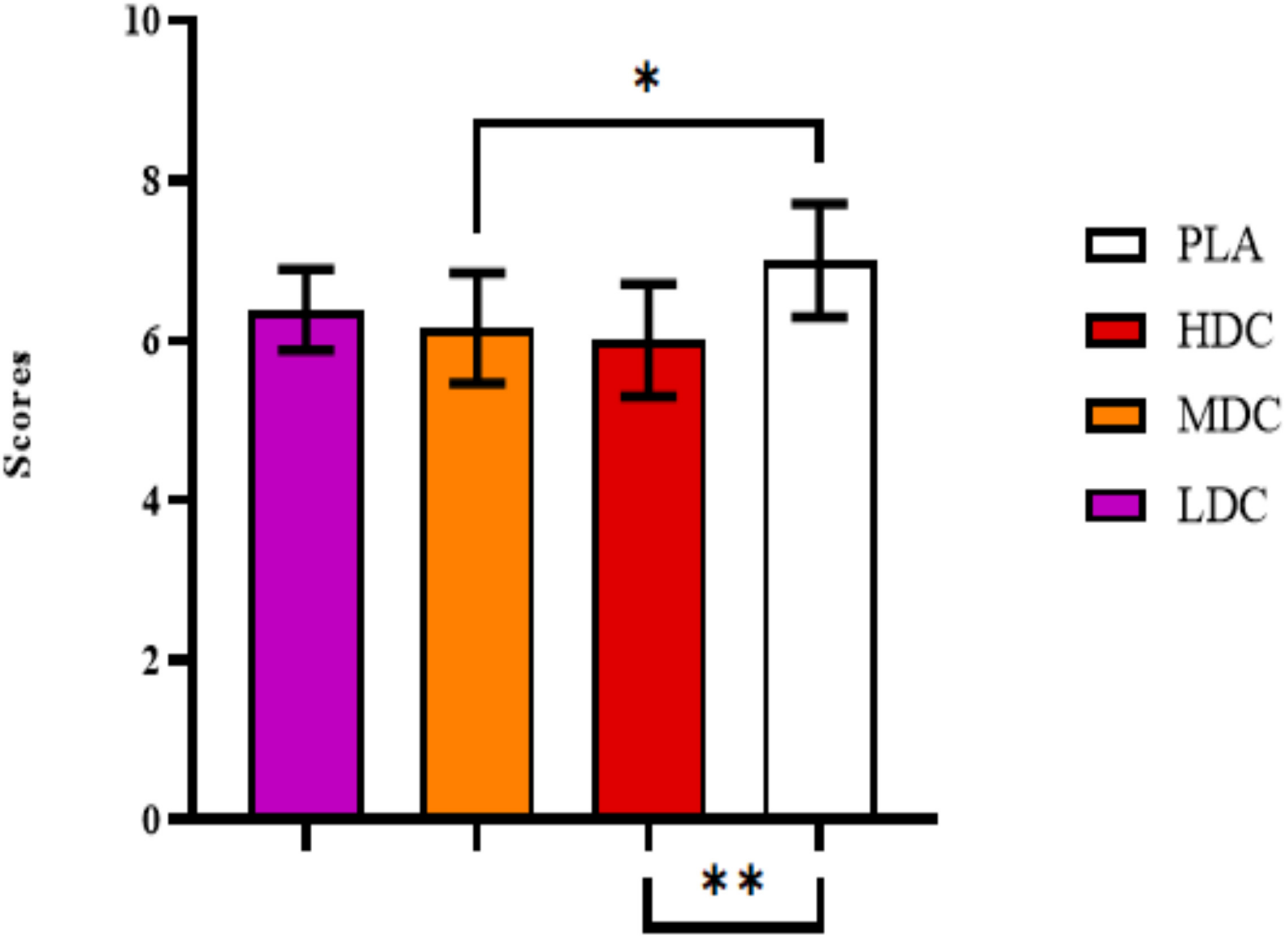
Subjective sleep quality scale values. PLA, Placebo; HDC, High Dose Caffeine; MDC, Moderate Dose Caffeine; LDC, Low Dose Caffeine. *p < 0.05, **p < 0.01.

Subjective sleep quality scale values have been provided in Figure 5.

There was a significant difference in sleep variables between conditions (*p* = 0.001, η*_*p*_*^2^ = 0.42). Bonferroni *post hoc* analysis showed worse sleep quality for HDC (6 ± 0.707) compared to PLA (7 ± 0.707, *p* = 0.005; 95% CI = −1.714 to 0.286; *d* = 1.50). Significantly worse sleep quality scores have been reported in MDC (6.154 ± 0.689) trial than PLA (7 ± 0.707, *p* = 0.032; 95% CI = −1.632 to −0.060; *d* = 1.30). On the other hand, participants in LDC trial reported worse sleep quality despite non-significance, values were close to significance (*p* = 0.082) and may have a practical effect during practice.

**FIGURE 6:**
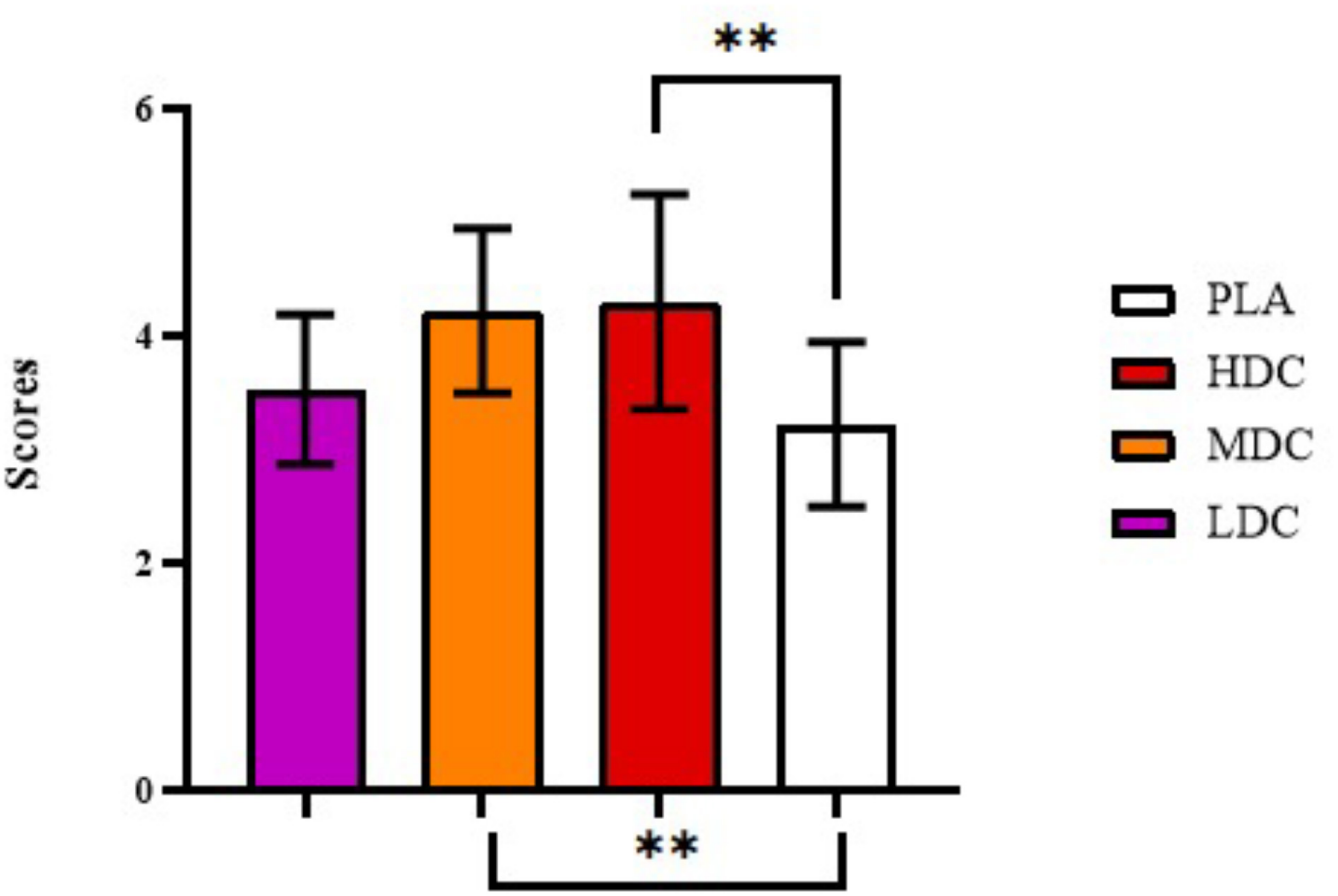
Karolinska daytime sleepiness scale values. PLA, Placebo; HDC, High Dose Caffeine; MDC, Moderate Dose Caffeine; LDC, Low Dose Caffeine. ** *p*< 0.01.

Karolinska daytime sleepiness scale values have been provided in Figure 6.

Significant differences in daytime sleepiness have been reported between conditions (*p* = 0.002, η*_*p*_*^2^ = 0.37). Daytime sleepiness values reported in HDC trial (4.308 ± 0.947) were significantly higher than PLA (3.231 ± 0.725, *p* = 0.009, 95% CI = 0.243–1.911; *d* = 1.40). Similarly, daytime sleepiness values reported during MDC trial (4.231 ± 0.725) were significantly higher than PLA (3.231 ± 0.725, *p* = 0.005, 95% CI = 0.286–1.714; *d* = 1.30).

Reported side effects after 24 h post-test was presented in Table 2.

**TABLE 2:** The incidence of side effects reported by participants (*n* = 13).

Variables	PLA	LDC	MDC	HDC	OR HDC vs. PLA (95% CI)	OR HDC vs. LDC (95% CI)	OR HDC vs. MDC (95% CI)
Increased urine output	0	1	2	5*#	∞ (3.82–∞)	5.49 (0.48–62.8)	2.72 (0.46–16.1)
Muscle soreness	1	1	2	2	2.11 (0.12–37.9)	2.11 (0.12–37.9)	1.00 (0.12–8.60)
Headache	0	0	1	5*#	∞ (3.41–∞)	∞ (1.31–∞)	5.44 (0.54–55.3)
Tachycardia	0	0	2	4*#	∞ (3.10–∞)	∞ (2.03–∞)	2.11 (0.35–12.8)
Increased vigor	0	0	1	2*#	∞ (1.31–∞)	∞ (1.31–∞)	2.11 (0.18–25.0)
Anxiety/nervousness	0	0	1	4*#	∞ (2.03–∞)	∞ (2.03–∞)	4.33 (0.46–40.6)
Gastrointestinal problems	0	0	1	6*#	∞ (4.78–∞)	∞ (4.78–∞)	6.54 (0.72–59.2)
Insomnia	0	2	5	7*#	∞ (4.33–∞)	3.74 (0.67–20.8)	1.47 (0.42–5.12)

PLA, Placebo; HDC, High Dose Caffeine, MDC, Moderate Dose Caffeine; LDC, Low Dose Caffeine. *p < 0.05 vs. PLA, #*p* < 0.05 vs. LDC, OR, Odd ratios, ∞, OR undefined due to zero incidence in reference group; lower bound estimated using Haldane–Anscombe continuity correction (add 0.5 to zero cells).

Cochran’s *Q*-test showed significant overall differences for urine output (*Q* = 11.6, *p* = 0.008), headache (*p* = 0.004), gastrointestinal problems (*p* = 0.002), and insomnia (*p* = 0.001). High-dose caffeine (HDC) significantly increased the incidence of increased urine output, headache, gastrointestinal problems, and insomnia compared to both placebo (*p* < 0.05) and low-dose caffeine (LDC) (*p* < 0.05, McNemar’s exact). Just three participants accurately discerned two out of the four experimental conditions, while another three correctly identified only one condition. Collectively, these results suggest that the randomization process was effective. Only one participant out of thirteen accurately identified the full set of experimental conditions, indicating that the blinding procedure was successful.

## 4 Discussion

The current study presents an experimental design that examines the effects of different doses of caffeine (low: LDC, medium: MDC, high: HDC) taken in the evening on timed rowing performance, cardiovascular responses, sleep quality, and daytime alertness. The main findings of the study showed that MDC and HDC in particular produced ergogenic effects in terms of performance, but these effects were accompanied by significant sleep disruptions and side effects with increasing doses.

Time-of-day performance differences are attracting the attention of various stakeholders in the field of sports science—particularly athletes, coaches, and researchers—as an important variable affecting performance. Studies focusing on performance evaluations conducted at different time periods are being designed to reveal intraday performance fluctuations. These studies can serve as scientific guides for planning performance-optimizing interventions. It has been reported in the literature that 15 active male participants performed better in the afternoon (17:00) compared to the morning session in the 5-meter multiple shuttles run test (60). In another study, when the effect of time of day on repeated sprint performance was examined, higher muscle strength was reported during the afternoon hours or early evening compared to the morning hours (61). Unlike these studies focusing on field-based sprint or agility performance, the present study specifically assessed time-trial performance over a fixed distance on a rowing ergometer. However, unlike the current research, these studies did not include measurements of performance against time for a fixed distance. Moreover, in a study conducted specifically on rowers, in which performances on the 2,000 m rowing ergometer in the morning and evening were evaluated, it was reported that they performed an average of 2.4 s faster in the morning training compared to the evening, regardless of chronotype (19). Considering current research, it is understandable that rowers need supplements to optimize their performance in the evening training. In the current study, where the test sessions were conducted in the evening, both the HDC (9 mg/kg) and MDC (6 mg/kg) conditions produced significantly shorter 2,000 m rowing ergometer completion times compared with PLA, indicating a dose-dependent improvement in performance. Although the difference was not statistically significant, the mean completion time in the LDC condition (405.715 ± 11.067 s) was still slightly faster than in the PLA condition (407.585 ± 10.615 s). Christensen et al. (62) demonstrated that even a difference of just 0.31% in average speed in Olympic endurance events can alter the medal standings. This finding is consistent with the fact that time differences among top-level athletes in international rowing competitions are often limited to just a few seconds (World Rowing, 2024; 2024a; 2025). Therefore, even if the approximately 2-s improvement observed in the LDC condition in the current study was not statistically significant, it still indicates a performance gain that could be of practical importance in a competitive environment. On the other hand, the minimum effective improvement of 0.70% reported by Grgic et al. (63) provides a meaningful reference point for interpreting the limited impact of low-dose caffeine on time-trial performance. In this context, the findings of our study provide important data regarding the practical importance of low-dose caffeine. Overall, the findings of the current study are consistent with previous studies on the effects of caffeine on short-term high-intensity performance (6, 39, 64, 65). Similar to our findings, Bruce et al. (39) in an experimental study in which diet and training were well controlled, it was observed that caffeine (6 or 9 mg/kg) consumed before a short-term high-intensity endurance test provided a significant increase in performance. In another study, 3 mg/kg caffeine intake in the evening did not significantly improve athletes’ 100 m swimming time trial performances (66). Doherty et al. (67) reported that caffeine had a more limited ergogenic effect in intense and short-term exercises compared to long-term endurance exercises. When the findings from the current study and the existing literature are considered together, it appears that higher caffeine doses are required to achieve measurable ergogenic benefits in short-distance time trials. Another reason why low-dose caffeine did not provide a significant improvement in rowing performance may be the daily caffeine consumption levels of the participants. The average daily caffeine consumption of the participants in the current study was 303.62 mg (SD = 148.34). This level of caffeine administration may have resulted in the low dose being lower than participants’ habitual intake, thus limiting the stimulatory effects of the low dose. It is known that regular caffeine intake can lead to tolerance by reducing the sensitivity of adenosine receptors (68, 69). Such tolerance may attenuate the stimulatory effects of low doses on the central nervous system and hinder the emergence of ergogenic responses. This mechanism partially explains why the more pronounced performance improvements observed in the high- and moderate-dose conditions in our study were limited in the low-dose condition. It may require higher caffeine doses to produce similar ergogenic responses in individuals with habitual high caffeine consumption. However, it is important to note that the effects of caffeine tolerance vary considerably among individuals (70). Studies also suggest that tolerance does not completely eliminate the performance-enhancing effects of caffeine and that even habitual users may experience significant benefits (71, 72). Power output is usually the primary performance measure for ergometer tests. It is essential to establish a reliable rowing performance test to test the effectiveness of an intervention on a rower’s power generation ability (73). The mean percentage standard error (%SEM) for mean power between repeated 2,000 m performances on the Concept II ergometer has been reported as 2.0% (55). In other words, it can be said that the 2.0% difference in mean power in repeated tests is due to natural measurement variability. In the present study, the 3.2% increase in mean power obtained in HDC and PLA conditions is above the 2.0% natural measurement variability. This supports the possibility that the observed difference is due to an ergogenic effect. There is also a significant difference between MDC and PLA. MDC supplementation resulted in a 2.45% increase in average strength compared to PLA. Similar to the HDC condition, this increase over the natural measurement variability supports the significant effect of the MDC intervention. The findings of the present study are consistent with the literature. In addition, a significant difference was found between the mean power outputs observed between HDC and LDC conditions in favor of HDC. The 1.75% difference between the mean power outputs suggests that the effect of high-dose caffeine intake on performance is dose-dependent, similar to our findings in time trial performance. Although the significant difference between HDC and LDC is within the natural measurement variability limit of the test, it has practical importance in time trial rowing. The findings of the present study align with previous research (39, 64, 74). In particular, the magnitude of the power improvements observed following caffeine ingestion closely mirrors the results reported by Bruce et al. (39), who documented a 2.7% increase in mean power during a 2,000 m rowing test in elite rowers. In another study evaluating the 100 m time trial performance of swimmers, 5 mg/kg caffeine intake shortened both the completion time and increased the average power production (3.6%) (74). As previously described, low-dose caffeine administration may have provided insufficient stimulation for the stimulant effect, as it was below the habitual daily consumption level of this group. This may be why no significant difference in mean power was observed in the LDC condition compared to placebo. Considering the time trial times and power outputs, it can be said that caffeine intake improves rowing performance. The caffeine-induced time-trial responses observed in our study parallel the dose-response profile of time trial performances reported in cyclists (75). Using completion time and average power output as key performance indicators, Chen et al. (75) meta-analysis reported that moderate caffeine doses (4–6 mg/kg), identified as the optimal range, significantly improved time trial performance, while lower doses (1–3 mg/kg) did not provide comparable benefits.

In the current study, when HDC and MDC caffeine intakes were compared with PLA, significant differences in mean heart rate were associated with small and moderate increases. It is known that the autonomic nervous system, which is managed by the central nervous system, plays an important role in controlling heart rate (76, 77). Considering the effect of caffeine on the central nervous system, it is reasonable that it plays a role in changes in heart rate (78). Accordingly, the heart rate increases observed in the present study under HDC and MDC conditions support the known sympathetic stimulatory effect of caffeine. Although an increase in heart rate during exercise is a physiologically expected result, this may lead to tolerance problems in some individuals during submaximal exercises. Contrary to the current research findings, Bruce et al. (39) did not observe a significant difference in heart rate during exercise after 6 and 9 mg/kg caffeine intake. The contradictory physiological responses to caffeine and exercise may be due to multiple factors, including timing of measurement, individual metabolic differences, and consumption habits (4, 46). Bruce et al. (39) did not provide information about when the performance test was initiated after caffeine intake. Therefore, similar results may not have been obtained due to the temporal difference between caffeine and performance testing.

Despite the potential of caffeine to enhance performance, sleep deprivation, which poses a risk of performance deficiencies, is among its known side effects (79, 80). Drake et al. (31) stated that even 400 mg of caffeine taken 6 h before bedtime significantly reduced sleep quality. Furthermore, it is known that caffeine (200 mg, 3+3 mg/kg, 6 mg/kg), especially in the afternoon, both before and without exercise, causes deterioration in sleep-related parameters (23–25). The findings of the current study, in which the test sessions started between 19:00 and 20:00, support the literature. Caffeine consumption was found to have a significantly deteriorating effect on sleep quality in the following night in HDC and MDC conditions compared to PLA. Although the pharmacokinetics of caffeine vary between individuals (81), it generally has a half-life ranging from 2 to 10 h (22). Although the plasma concentration of caffeine decreases over time, it is likely that it will continue to have a stimulating effect until it is completely eliminated, thus negatively affecting sleep quality. Although sleep quality was assessed only via a scale in the current study, Ali et al. (82) showed that VO2, RER, and heart rate values measured during sleep remained high after caffeine intake. These findings support the idea that caffeine may impair sleep quality by maintaining metabolic stimulation even during sleep. In addition, although the deterioration in sleep quality in LDC did not reach statistical significance in our study, the trend was negative. Although caffeine taken in the evening can disrupt all types of sleep parameters through 6-sulfatoxymelatonin (the main metabolite of melatonin) (83) and adrenaline and noradrenaline stimulation in the adrenal medulla (84, 85), the majority of sleep disruptions are thought to be dose-dependent (86). Karacan et al. (87) reported that among the caffeine doses given 30 min before bedtime, caffeine equivalent to 4 cups of coffee disrupted total sleep time, emphasizing that the effect was dose-related. In parallel with our findings, a study conducted with highly trained judokas reported that low-dose (3 mg/kg) caffeine intake administered before evening training did not cause a significant deterioration in objective sleep quality (88). In contrast, Miller et al. (24) concluded that 3 mg/kg caffeine caused significant sleep disturbance, but participants did not perform any exercise after caffeine ingestion. Moreover, it is known that acute exercise improves both sleep quality and sleep latency (89, 90). In the current study, the negative effect of low-dose caffeine on sleep disruption may have been inhibited thanks to this known benefit of exercise.

When the daytime sleepiness data are examined, it is notable that both the HDC and MDC conditions show significantly higher sleepiness levels compared to PLA. This observed increase may be associated with the decrease in the quality of nighttime sleep at the same doses. Laboratory studies show that an average decrease of 90 min in nighttime sleep can lead to a decrease in objective alertness the next day by approximately one-third (91). Moreover, sleep deprivation causes significant decreases in cognitive functions such as cognitive and psychomotor reaction speed in the following days (92). This may be a limiting factor in terms of the sustainability of performance. It has been reported that even one night of disrupted sleep negatively affects performance in the next competition or training (93, 94). Therefore, it is important to be aware of the potential risks of caffeine use, which is well known to have negative effects on sleep. Additionally, no significant changes in daytime sleepiness were observed following LDC supplementation compared to the other conditions. Given that daytime sleepiness is linked to nighttime sleepiness, the lack of statistical significance in the LDC condition is consistent with the findings of the current study regarding sleep quality.

Lastly, when evaluated in terms of side effects, the current study reported that in addition to insomnia after the HDC condition, effects such as headache, gastrointestinal problems and frequent urination increased significantly. Although side effects were generally reported less frequently in the MDC condition than in the HDC condition, the highest number of side effects was reported in the insomnia variable, with 5 participants. No significant increase was found in the LDC condition. In support of our results, Pallares et al. (35) study, which examined the side effects of three caffeine doses, also found a significant increase in the frequency of side effects at a 9 mg/kg dose compared to the other conditions. Furthermore, while some side effects were observed in the MDC condition, they were lower than the side effect rate reported after HDC. In the LDC condition, the side effect rate was similar to that reported after PLA. Another study found no significant difference in self-reported physical or psychological side effects of 3 mg/kg caffeine intake compared to placebo (66). Therefore, these known side effects of HDC outweigh its positive performance effects. The current study provides evidence supporting the recommended caffeine dose of 3–6 mg/kg.

The current study has several strengths. It is one of the few studies that examines the effects of pre-exercise caffeine use on both performance and sleep. Furthermore, it is one of the studies that can lead to the determination of the most appropriate supplementation amount by comparing different doses. Reliable and reproducible data were provided through the analyses. However, certain limitations should be acknowledged. Since plasma caffeine concentrations were not measured, it was not possible to determine to what extent the observed sleep disturbances were related to this variable. Additionally, sleep parameters were assessed only through self-reporting. However, individual differences could not be taken into account because the participants’ habitual sleep patterns were not monitored. A further limitation is that participants’ baseline physiological or psychological state prior to each trial were not directly assessed. Although habitual routines were monitored and subjective sleep and sleepiness ratings were obtained after each session, the absence of immediate pre-trial baseline measures may have introduced variability across sessions. Finally, the sample group consisting of trained individuals with moderate caffeine consumption habits limits its generalizability to different training and caffeine sensitivity levels. Future research should consider incorporating repeated measurements at multiple time points within each trial to allow for the assessment of potential caffeine dose × time interaction effects, thereby providing a more detailed understanding of how ergogenic and physiological responses to caffeine may vary throughout the course of a session.

## 5 Conclusion

In conclusion, evening caffeine intake demonstrated a dose-dependent effect, with both medium (6 mg/kg) and high doses (9 mg/kg) improving rowing performance. However, these benefits were accompanied by dose-related side effects, particularly sleep disruption and increased adverse events at higher doses. Among the tested protocols, the medium dose (6 mg/kg) appears to provide the most balanced option, enhancing performance while limiting negative outcomes. Although the low dose (3 mg/kg) did not lead to statistically significant improvements, the observed reduction in time trial completion (≈2 s) may hold practical relevance in competitive settings where marginal gains are critical. These findings highlight the importance of considering both ergogenic benefits and potential drawbacks when determining practical caffeine supplementation strategies for athletes.

## Data Availability

The raw data supporting the conclusions of this article will be made available by the authors upon reasonable request. Further inquiries can be directed to the corresponding author.

## References

[1] Pickering C Grgic J. Is coffee a useful source of caffeine preexercise? *Int J Sport Nutr Exerc Metab.* (2020) 30:69–82. 10.1123/ijsnem.2019-0092 31629349

[2] Del Coso J Muñoz G Muñoz-Guerra J. Prevalence of caffeine use in elite athletes following its removal from the world anti-doping agency list of banned substances. *Appl Physiol Nutr Metab.* (2011) 36:555–61. 10.1139/h11-052 21854160

[3] Van Thuyne W Roels K Delbeke F. Distribution of caffeine levels in urine in different sports in relation to doping control. *Int J Sports Med.* (2005) 26:714–8. 10.1055/s-2005-837437 16237615

[4] Aguilar-Navarro M Muñoz G Salinero JJ Muñoz-Guerra J Fernández-Álvarez M Plata MDM Urine caffeine concentration in doping control samples from 2004 to 2015. *Nutrients.* (2019) 11:286. 10.3390/nu11020286 30699902 PMC6412495

[5] Wickham KA Spriet LL. Administration of caffeine in alternate forms. *Sports Med.* (2018) 48:79–91. 10.1007/s40279-017-0848-2 29368182 PMC5790855

[6] Southward K Rutherfurd-Markwick KJ Ali A. The effect of acute caffeine ingestion on endurance performance: a systematic review and meta–analysis. *Sports Med.* (2018) 48:1913–28. 10.1007/s40279-018-0939-8 29876876

[7] Grgic J. Caffeine ingestion enhances Wingate performance: a meta-analysis. *Eur J Sport Sci.* (2018) 18:219–25. 10.1080/17461391.2017.1394371 29087785

[8] Raya-Gonzalez J Rendo-Urteaga T Domínguez R Castillo D Rodriguez-Fernandez A Grgic J. Acute effects of caffeine supplementation on movement velocity in resistance exercise: a systematic review and meta-analysis. *Sports Med.* (2020) 50:717–29. 10.1007/s40279-019-01211-9 31643020

[9] Grgic J Trexler ET Lazinica B Pedisic Z. Effects of caffeine intake on muscle strength and power: a systematic review and meta-analysis. *J Int Soc Sports Nutr.* (2018) 15:11. 10.1186/s12970-018-0216-0 29527137 PMC5839013

[10] Polito M Souza D Casonatto J Farinatti P. Acute effect of caffeine consumption on isotonic muscular strength and endurance: a systematic review and meta-analysis. *Sci Sports.* (2016) 31:119–28. 10.1016/j.scispo.2016.01.006

[11] Fredholm BB. Adenosine, adenosine receptors and the actions of caffeine. *Pharmacol Toxicol.* (1995) 76:93–101. 10.1111/j.1600-0773.1995.tb00111.x 7746802

[12] Astorino TA Roberson DW. Efficacy of acute caffeine ingestion for short-term high-intensity exercise performance: a systematic review. *J Strength Condition Res.* (2010) 24:257–65. 10.1519/JSC.0b013e3181c1f88a 19924012

[13] Guest N Corey P Vescovi J El-Sohemy A. Caffeine, CYP1A2 genotype, and endurance performance in athletes. *Med Sci Sports Exerc.* (2018) 50:1570–8. 10.1249/MSS.0000000000001596 29509641

[14] Higgins S Straight CR Lewis RD. The effects of preexercise caffeinated coffee ingestion on endurance performance: an evidence-based review. *Int J Sport Nutr Exerc Metab.* (2016) 26:221–39. 10.1123/ijsnem.2015-0147 26568580

[15] Mohr M Nielsen JJ Bangsbo J. Caffeine intake improves intense intermittent exercise performance and reduces muscle interstitial potassium accumulation. *J Appl Physiol.* (2011) 111:1372–9. 10.1152/japplphysiol.01028.2010 21836046

[16] Kalmar JM Cafarelli E. Caffeine: a valuable tool to study central fatigue in humans? *Exerc Sport Sci Rev.* (2004) 32:143–7. 10.1097/00003677-200410000-00004 15604932

[17] Spriet LL. Exercise and sport performance with low doses of caffeine. *Sports Med.* (2014) 44:175–84. 10.1007/s40279-014-0257-8 25355191 PMC4213371

[18] Steinacker JM. Physiological aspects of training in rowing. *Int J Sports Med.* (1993) 14:S3–3. 8262704

[19] Brown FM Neft EE LaJambe CM. Collegiate rowing crew performance varies by morningness-eveningness. *J Strength Condition Res.* (2008) 22:1894–900. 10.1519/JSC.0b013e318187534c 18978619

[20] Wikoff D Welsh BT Henderson R Brorby GP Britt J Myers E Systematic review of the potential adverse effects of caffeine consumption in healthy adults, pregnant women, adolescents, and children. *Food Chem Toxicol.* (2017) 109:585–648. 10.1016/j.fct.2017.04.002 28438661

[21] de Souza JG Del Coso J Fonseca FDS Silva BVC de Souza DB da Silva Gianoni RL Risk or benefit? Side effects of caffeine supplementation in sport: a systematic review. *Eur J Nutr.* (2022) 61:3823–34. 10.1007/s00394-022-02874-3 35380245

[22] Snel J Lorist MM. Effects of caffeine on sleep and cognition. *Prog Brain Res.* (2011) 190:105–17. 10.1016/B978-0-444-53817-8.00006-2 21531247

[23] Drapeau C Hamel-Hébert I Robillard R Selmaoui B Filipini D Carrier J. Challenging sleep in aging: the effects of 200 mg of caffeine during the evening in young and middle-aged moderate caffeine consumers. *J Sleep Res.* (2006) 15:133–41. 10.1111/j.1365-2869.2006.00518.x 16704567

[24] Miller B O’Connor H Orr R Ruell P Cheng HL Chow CM. Combined caffeine and carbohydrate ingestion: effects on nocturnal sleep and exercise performance in athletes. *Eur J Appl Physiol.* (2014) 114:2529–37. 10.1007/s00421-014-2973-z 25115507

[25] Ramos-Campo DJ Pérez A Ávila-Gandía V Pérez-Piñero S Rubio-Arias J. Impact of caffeine intake on 800-m running performance and sleep quality in trained runners. *Nutrients.* (2019) 11:2040. 10.3390/nu11092040 31480553 PMC6770771

[26] Bonnar D Bartel K Kakoschke N Lang C. Sleep interventions designed to improve athletic performance and recovery: a systematic review of current approaches. *Sports Med.* (2018) 48:683–703. 10.1007/s40279-017-0832-x 29352373

[27] Fullagar HH Skorski S Duffield R Hammes D Coutts AJ Meyer T. Sleep and athletic performance: the effects of sleep loss on exercise performance, and physiological and cognitive responses to exercise. *Sports Med.* (2015) 45:161–86. 10.1007/s40279-014-0260-0 25315456

[28] Caia J Halson SL Holmberg PM Kelly VG. Does caffeine consumption influence postcompetition sleep in professional rugby league athletes? A case study. *Int J Sports Physiol Perform.* (2021) 17:126–9. 10.1123/ijspp.2020-0841 34340214

[29] Dunican IC Higgins CC Jones MJ Clarke MW Murray K Dawson B Caffeine use in a super rugby game and its relationship to post-game sleep. *Eur J Sport Sci.* (2018) 18:513–23. 10.1080/17461391.2018.1433238 29431593

[30] Burke LM. Caffeine and sports performance. *Appl Physiol Nutr Metab.* (2008) 33:1319–34. 10.1139/H08-130 19088794

[31] Drake C Roehrs T Shambroom J Roth T. Caffeine effects on sleep taken 0, 3, or 6 hours before going to bed. *J Clin Sleep Med.* (2013) 9:1195–200. 10.5664/jcsm.3170 24235903 PMC3805807

[32] McHill AW Smith BJ Wright KP. Effects of caffeine on skin and core temperatures, alertness, and recovery sleep during circadian misalignment. *J Biol Rhythms.* (2014) 29:131–43. 10.1177/07487304145230 24682207

[33] Clarke N Baxter H Fajemilua E Jones V Oxford S Richardson D Coffee and caffeine ingestion have little effect on repeated sprint cycling in relatively untrained males. *Sports.* (2016) 4:45. 10.3390/sports4030045 29910293 PMC5968880

[34] Sabblah S Dixon D Bottoms L. Sex differences on the acute effects of caffeine on maximal strength and muscular endurance. *Comp Exerc Physiol.* (2015) 11:89–94. 10.3920/CEP150010 29510743

[35] Pallares JG Fernandez-Elias VE Ortega JF Munoz G Munoz-Guerra J Mora-Rodriguez R. Neuromuscular responses to incremental caffeine doses: performance and side effects. *Med Sci Sports Exerc.* (2013) 45:2184–92. 10.1249/MSS.0b013e31829a6672 23669879

[36] Wilk M Krzysztofik M Filip A Zajac A Del Coso J. The effects of high doses of caffeine on maximal strength and muscular endurance in athletes habituated to caffeine. *Nutrients.* (2019) 11:1912. 10.3390/nu11081912 31443220 PMC6722777

[37] Guest NS VanDusseldorp TA Nelson MT Grgic J Schoenfeld BJ Jenkins ND International society of sports nutrition position stand: caffeine and exercise performance. *J Int Soc Sports Nutr.* (2021) 18:1. 10.1186/s12970-020-00383-4 33388079 PMC7777221

[38] Karayigit R Naderi A Akca F Cruz CJGD Sarshin A Yasli BC Effects of different doses of caffeinated coffee on muscular endurance, cognitive performance, and cardiac autonomic modulation in caffeine naive female athletes. *Nutrients.* (2020) 13:2. 10.3390/nu13010002 33374947 PMC7821939

[39] Bruce CR Anderson ME Fraser SF Stepto NK Klein R Hopkins WG Enhancement of 2000-m rowing performance after caffeine ingestion. *Med Sci Sports Exerc.* (2000) 32:1958–63. 10.1097/00005768-200011000-00021 11079528

[40] Gharaat MA Sheykhlouvand M Eidi LA. Performance and recovery: effects of caffeine on a 2000-m rowing ergometer. *Sport Sci Health.* (2020) 16:531–42. 10.1007/s11332-020-00643-5

[41] Skinner TL Jenkins DG Coombes JS Taaffe DR Leveritt MD. Dose response of caffeine on 2000-m rowing performance. *Med Sci Sports Exerc.* (2010) 42:571–6. 10.1249/mss.0b013e3181b6668b 19952822

[42] Filip-Stachnik A Krawczyk R Krzysztofik M Rzeszutko-Belzowska A Dornowski M Zajac A Effects of acute ingestion of caffeinated chewing gum on performance in elite judo athletes. *J Int Soc Sports Nutr.* (2021) 18:49. 10.1186/s12970-021-00448-y 34147116 PMC8214258

[43] Burke LM. Practical issues in evidence-based use of performance supplements: supplement interactions, repeated use and individual responses. *Sports Med.* (2017) 47:79–100. 10.1007/s40279-017-0687-1 28332111 PMC5371635

[44] Urbaniak GC Plous S. *Research Randomizer (Version 4.0).* (2013). Available online at: http://www.randomizer.org/

[45] Gardiner CL Weakley J Burke LM Fernandez F Johnston RD Leota J Dose and timing effects of caffeine on subsequent sleep: a randomized clinical crossover trial. *Sleep.* (2025) 48:zsae230. 10.1093/sleep/zsae230 39377163 PMC11985402

[46] Pickering C Kiely J. What should we do about habitual caffeine use in athletes? *Sports Med.* (2019) 49:833–42. 10.1007/s40279-018-0980-7 30173351 PMC6548063

[47] Cohen J. *Statistical Power Analysis For the Behavioral Sciences.* Milton Park: Routledge (2013).

[48] Grgic J Diaz-Lara FJ Del Coso J Duncan MJ Tallis J Pickering C The effects of caffeine ingestion on measures of rowing performance: a systematic review and meta-analysis. *Nutrients.* (2020) 12:434. 10.1007/s40279-019-01250-8 32046330 PMC7071243

[49] Flueck JL Schaufelberger F Lienert M Schäfer Olstad D Wilhelm M Perret C. Acute effects of caffeine on heart rate variability, blood pressure and tidal volume in paraplegic and tetraplegic compared to able-bodied individuals: a randomized, blinded trial. *PLoS One.* (2016) 11:e0165034. 10.1371/journal.pone.0165034 27776149 PMC5077167

[50] Bühler E Lachenmeier DW Schlegel K Winkler G. Development of a tool to assess the caffeine intake among teenagers and young adults. *Ernahrungs Umschau.* (2014) 61:58–63. 10.4455/eu.2014.011

[51] Filip A Wilk M Krzysztofik M Del Coso J. Inconsistency in the ergogenic effect of caffeine in athletes who regularly consume caffeine: is it due to the disparity in the criteria that defines habitual caffeine intake? *Nutrients.* (2020) 12:1087. 10.3390/nu12041087 32326386 PMC7230656

[52] Tallis J Clarke N Morris R Richardson D Ellis M Eyre E The prevalence and practices of caffeine use as an ergogenic aid in English professional soccer. *Biol Sport.* (2021) 38:525–34. 10.5114/biolsport.2021.101125 34937961 PMC8670797

[53] Snyder E Cai B DeMuro C Morrison MF Ball W. A new single-item sleep quality scale: results of psychometric evaluation in patients with chronic primary insomnia and depression. *J Clin Sleep Med.* (2018) 14:1849–57. 10.5664/jcsm.7478 30373688 PMC6223557

[54] Shahid A Wilkinson K Marcu S Shapiro CM. Time of day sleepiness scale (TODSS). In: Shahid A Wilkinson K Marcu S Shapiro CM editors. *STOP, THAT and One Hundred Other Sleep Scales.* New York, NY: Springer (2012). p. 389–90. 10.1007/978-1-4419-9893-4_95

[55] Schabort E Hawley J Hopkins W Blum H. High reliability of performance of well-trained rowers on a rowing ergometer. *J Sports Sci.* (1999) 17:627–32. 10.1080/026404199365650 10487463

[56] O’Neill T Skelton A. *Indoor Rowing Training Guide.* Morrisville, VT: Concept 2 Ltd (2004). 2004 p.

[57] Hahn A Bourdon P Tanner R. *Protocols For the Physiological Assessment of Rowers. Physiological Tests for Elite Athletes.* Champaign, IL: Human Kinetics (2000). p. 311–26.

[58] Pripstein LP Rhodes EC McKenzie DC Coutts KD. Aerobic and anaerobic energy during a 2-km race simulation in female rowers. *Eur J Appl Physiol Occup Physiol.* (1999) 79:491–4. 10.1007/s004210050542 10344457

[59] Cohen J. A power primer. *Psychol Bull.* (1992) 112:155. 10.1037/0033-2909.112.1.155 19565683

[60] Souissi Y Souissi M Chtourou H. Effects of caffeine ingestion on the diurnal variation of cognitive and repeated high-intensity performances. *Pharmacol Biochem Behav.* (2019) 177:69–74. 10.1016/j.pbb.2019.01.001 30611752

[61] Aloui A Chaouachi A Chtourou H Wong DP Haddad M Chamari K Effects of Ramadan on the diurnal variations of repeated-sprint performance. *Int J Sports Physiol Perform.* (2013) 8:254–63. 10.1123/ijspp.8.3.254 22952200

[62] Christensen PM Shirai Y Ritz C Nordsborg NB. Caffeine and bicarbonate for speed. a meta-analysis of legal supplements potential for improving intense endurance exercise performance. *Front Physiol.* (2017) 8:240. 10.3389/fphys.2017.00240 28536531 PMC5422435

[63] Grgic J Grgic I Pickering C Schoenfeld BJ Bishop DJ Pedisic Z. Wake up and smell the coffee: caffeine supplementation and exercise performance–an umbrella review of 21 published meta-analyses. *Br J Sports Med.* (2020) 54:681–8. 10.1136/bjsports-2018-100278 30926628

[64] Anderson ME Bruce CR Fraser SF Stepto NK Klein R Hopkins WG Improved 2000-meter rowing performance in competitive oarswomen after caffeine ingestion. *Int J Sport Nutr Exerc Metab.* (2000) 10:464–75. 10.1123/ijsnem.10.4.464 11099373

[65] Davis J-K Green JM. Caffeine and anaerobic performance: ergogenic value and mechanisms of action. *Sports Med.* (2009) 39:813–32. 10.2165/11317770-000000000-00000 19757860

[66] Newbury JW Saunders B Gough LA. Evening caffeine did not improve 100-m swimming time trials performed 60 min post-ingestion or the next morning after sleep. *Int J Sport Nutr Exerc Metab.* (2022) 32:453–61. 10.1123/ijsnem.2022-0042 35894958

[67] Doherty M Smith PM Hughes MG Davison RR. Caffeine lowers perceptual response and increases power output during high-intensity cycling. *J Sports sci.* (2004) 22:637–43. 10.1080/02640410310001655741 15370494

[68] Harland BF. Caffeine and nutrition. *Nutrition.* (2000) 16:522–6. 10.1016/s0899-900700369-5 10906543

[69] Van Soeren M Graham T. Effect of caffeine on metabolism, exercise endurance, and catecholamine responses after withdrawal. *J Appl Physiol.* (1998) 85:1493–501. 10.1152/jappl.1998.85.4.1493 9760346

[70] Armstrong LE. Caffeine, body fluid-electrolyte balance, and exercise performance. *Int J Sport Nutr Exerc Metab.* (2002) 12:189–206. 10.1123/ijsnem.12.2.189 12187618

[71] Chesley A Howlett RA Heigenhauser GJ Hultman E Spriet LL. Regulation of muscle glycogenolytic flux during intense aerobic exercise after caffeine ingestion. *Am J Physiol Regul Integr Comp Physiol.* (1998) 275:R596–603. 10.1152/ajpregu.1998.275.2.R596 9688698

[72] Tarnopolsky M Cupido C. Caffeine potentiates low frequency skeletal muscle force in habitual and nonhabitual caffeine consumers. *J Appl Physiol.* (2000) 89:1719–24. 10.1152/jappl.2000.89.5.1719 11053318

[73] Soper C Hume PA. Rowing: reliability of power output during rowing changes with ergometer type and race distance. *Sports Biomech.* (2004) 3:237–48. 10.1080/14763140408522843 15552583

[74] Wiles JD Coleman D Tegerdine M Swaine IL. The effects of caffeine ingestion on performance time, speed and power during a laboratory-based 1 km cycling time-trial. *J Sports Sci.* (2006) 24:1165–71. 10.1080/02640410500457687 17035165

[75] Chen B Ding L Qin Q Lei T-H Girard O Cao Y. Effect of caffeine ingestion on time trial performance in cyclists: a systematic review and meta-analysis. *J Int Soc Sports Nutr.* (2024) 21:2363789. 10.1080/15502783.2024.2363789 38836626 PMC11155427

[76] Ginty AT Kraynak TE Fisher JP Gianaros PJ. Cardiovascular and autonomic reactivity to psychological stress: neurophysiological substrates and links to cardiovascular disease. *Auton Neurosci.* (2017) 207:2–9. 10.1016/j.autneu.2017.03.003 28391987 PMC5600671

[77] Quinlan P Lane J Aspinall L. Effects of hot tea, coffee and water ingestion on physiological responses and mood: the role of caffeine, water and beverage type. *Psychopharmacology.* (1997) 134:164–73. 10.1007/s002130050438 9399380

[78] Benjamim CJR Kliszczewicz B Garner DM Cavalcante TCF da Silva AAM Santana MDR Is caffeine recommended before exercise? A systematic review to investigate its impact on cardiac autonomic control via heart rate and its variability. *J Am Coll Nutr.* (2020) 39:563–73. 10.1080/07315724.2019.1705201 31860391

[79] Hindmarch I Rigney U Stanley N Quinlan P Rycroft J Lane J. A naturalistic investigation of the effects of day-long consumption of tea, coffee and water on alertness, sleep onset and sleep quality. *Psychopharmacology.* (2000) 149:203–16. 10.1007/s002130000383 10823400

[80] Youngstedt SD O’Connor PJ Crabbe JB Dishman RK. The influence of acute exercise on sleep following high caffeine intake. *Physiol Behav.* (2000) 68:563–70. 10.1016/S0031-938400213-9 10713298

[81] Temple JL Bernard C Lipshultz SE Czachor JD Westphal JA Mestre MA. The safety of ingested caffeine: a comprehensive review. *Front Psychiatry.* (2017) 8:80. 10.3389/fpsyt.2017.00080 28603504 PMC5445139

[82] Ali A O’Donnell J Starck C Rutherfurd-Markwick K. The effect of caffeine ingestion during evening exercise on subsequent sleep quality in females. *Int J Sports Med.* (2015) 36:433–9. 10.1055/s-0034-1398580 25700100

[83] Shilo L Sabbah H Hadari R Kovatz S Weinberg U Dolev S The effects of coffee consumption on sleep and melatonin secretion. *Sleep Med.* (2002) 3:271–3. 10.1016/S1389-945700015-1 14592218

[84] Sawyer DA Julia HL Turin AC. Caffeine and human behavior: arousal, anxiety, and performance effects. *J Behav Med.* (1982) 5:415–39. 10.1007/BF00845371 7154064

[85] Zhang Y-H Li Y-F Wang Y Tan L Cao Z-Q Xie C Identification and characterization of N 9-methyltransferase involved in converting caffeine into non-stimulatory theacrine in tea. *Nat Commun.* (2020) 11:1473. 10.1038/s41467-020-15324-7 32193380 PMC7081346

[86] Robillard R Bouchard M Cartier A Nicolau L Carrier J. Sleep is more sensitive to high doses of caffeine in the middle years of life. *J psychopharmacol.* (2015) 29:688–97. 10.1177/02698811155755 25759402

[87] Karacan I Thornby JI Anch AM Booth GH Williams RL Salis PJ. Dose-related sleep disturbances induced by coffee and caffeine. *Clin Pharmacol Therap.* (1976) 20:682–9. 10.1002/cpt1976206682 186223

[88] Filip-Stachnik A. Does acute caffeine intake before evening training sessions impact sleep quality and recovery-stress state? preliminary results from a study on highly trained judo athletes. *Appl Sci.* (2022) 12:9957. 10.3390/app12199957

[89] Driver H Taylor S. Exercise and sleep. *Sleep Med Rev.* (2000) 4:387–402. 10.1053/smrv.2000.0110 12531177

[90] Youngstedt SD. Effects of exercise on sleep. *Clin Sports Med.* (2005) 24:355–65. 10.1016/j.csm.2004.12.003 15892929

[91] Bonnet MH Arand DL. Hyperarousal and insomnia: state of the science. *Sleep Med Rev.* (2010) 14:9–15. 10.1016/j.smrv.2009.05.002 19640748

[92] Goel N Rao H Durmer JS Dinges DF. Neurocognitive consequences of sleep deprivation. *Semin Neurol.* (2009) 25:117–29. 10.1055/s-2005-867080 19742409 PMC3564638

[93] Oliver SJ Costa RJS Laing SJ Bilzon JLJ Walsh NP. One night of sleep deprivation decreases treadmill endurance performance. *Eur J Appl Physiol.* (2009) 107:155–61. 10.1007/s00421-009-1103-9 19543909

[94] Skein M Duffield R Edge J Short MJ Mündel T. Intermittent-sprint performance and muscle glycogen after 30 h of sleep deprivation. *Med Sci Sports Exerc.* (2011) 43:1301–11. 10.1249/MSS.0b013e31820abc5a 21200339

